# Draft Genome Sequence of *Methylobacterium radiotolerans* Strain MAMP 4754, a Bacterial Endophyte Isolated from *Combretum erythrophyllum* in South Africa

**DOI:** 10.1128/genomeA.00976-17

**Published:** 2017-10-05

**Authors:** Mampolelo M. Photolo, Vuyo Mavumengwana, Mahloro H. Serepa-Dlamini, Matsobane G. Tlou

**Affiliations:** a Department of Biochemistry, Faculty of Science, University of Johannesburg, Auckland Park Campus, Johannesburg, South Africa; b Department of Biotechnology and Food Technology, Faculty of Science, University of Johannesburg, Doornfontein Campus, Johannesburg, South Africa

## Abstract

We announce here the draft genome sequence of *Methylobacterium radiotolerans* strain MAMP 4754, isolated from the roots of the medicinal plant *Combretum erythrophyllum*. *M. radiotolerans* has a genome size of 7,389,282 bp with 7,166 genes and a G+C content of 70.5%.

## GENOME ANNOUNCEMENT

The genus *Methylobacterium* consists of pink-pigmented facultative methylotrophic bacterial species that are ubiquitous on the surface and the interior of plants (1, 2). Members of the genus have a beneficial association with the plant and have been reported to fix nitrogen (3, 4), produce cellulase (5), and interact with other plant pathogens by inducing systemic resistance (2, 6, 7). A wide variety of *Methylobacterium* spp. have been previously isolated from plants (2–4, 8–11). *M. radiotolerans* strain MAMP 4754 was isolated from the roots of the medicinal plant *Combretum erythrophyllum*.

The complete sequencing of strain MAMP 4754 will enrich the genome sequence database of *M. radiotolerans* and further our understanding of the genetic and phylogenetic properties of this strain and its association with plants. Here, we present the description of the draft genome sequence and the annotation of *M. radiotolerans* strain MAMP 4754, isolated from *C. erythrophyllum* according to methods described by Jasim et al. (12).

Genomic DNA was extracted from overnight solid bacterial colony cultures using a DNeasy blood and tissue kit (Qiagen, Germany). The extracted DNA was quantified using a NanoDrop ND-2000 UV-Vis spectrophotometer (Thermo Fisher Scientific, USA). Genomic DNA was fragmented (500 to 1,000 bp) using an ultrasonicator (Covaris, USA). The fragmented DNA was then end repaired and adaptor ligated using the NEBNext Ultra II DNA library prep kit for Illumina (New England Biolabs, USA). Library concentration was measured using a NEBnext Library Quant Kit (New England Biolabs) and quality validated using an Agilent 2100 bioanalyzer (Agilent Technologies, USA). The samples were pooled in equimolar concentrations and diluted to 4 nM based on library concentration and calculated library fragment size. The library pool was sequenced on the MiSeq platform (Illumina, USA) using a MiSeq PE V3 reagent kit (600-cycle; Illumina). The final pooled library was at 10 pM with 10% PhiX as the control. The data generated comprised 2 × 300-bp paired-end reads. Quality adapter trimming was performed on CLC Genomics Workbench version 10 (CLC Bio, Denmark).

A total of 2,239,502 paired-end reads at 192× coverage were obtained from this workflow. The genome was assembled using the *de novo* assembly tool from CLC Bio, which produced 1,724 contigs with an average length of 4,523 bp and an *N*_50_ value of 84,897 bp. The genome of *M. radiotolerans* strain MAMP 4754 has 7,389,282 bp, with a G+C content of 70.5%, which is similar to other *Methylobacterium* spp. (13, 14). Genome annotation was performed using the NCBI Prokaryotic Genome Annotation Pipeline. The *M. radiotolerans* strain MAMP 4754 genome has 7,166 genes, among which are 7,105 protein-coding sequence genes and 729 pseudogenes. Furthermore, the genome has 3 rRNAs (5S, 16S, and 23S), 53 tRNAs, and 5 noncoding RNAs. We found 34 genes that show homology to known genes, which are putatively responsible for the strain’s endophytic behavior.

### Accession number(s).

This whole-genome shotgun project has been deposited at DDBJ/ENA/GenBank under the accession number NKQS00000000. The version described in this paper is the first version, NKQS01000000.
